# Targeting Aurora-A inhibits tumor progression and sensitizes thyroid carcinoma to Sorafenib by decreasing PFKFB3-mediated glycolysis

**DOI:** 10.1038/s41419-023-05709-z

**Published:** 2023-03-29

**Authors:** Zhi Jingtai, Hu Linfei, Qian Yuyang, Kang Ning, Yun Xinwei, Wang Xin, Ruan Xianhui, Huang Dongmei, Yang Weiwei, Meng Xiangrui, Zhu Tianze, Wang Wei, Zheng Xiangqian

**Affiliations:** 1 Department of Thyroid and Neck Tumor, Tianjin Medical University Cancer Institute and Hospital, National Clinical Research Center for Cancer, Key Laboratory of Cancer Prevention and Therapy, Tianjin’s Clinical Research Center for Cancer, Tianjin, 300060 People’s Republic of China; 2 Department of Otorhinolaryngology, Head and Neck Surgery, Tianjin First Central Hospital, Institute of Otolaryngology of Tianjin, Key Laboratory of Auditory Speech and Balance Medicine, Key Medical Discipline of Tianjin (Otolaryngology), Quality Control Centre of Otolaryngology, Rehabilitation Road No.24, 300192 Tianjin, People’s Republic of China; 3 State Key Laboratory of Medicinal Chemical Biology and College of Life Sciences, Nankai University, 300071 Tianjin, China; 4 Department of Clinical Medicine, The Clinical Medicine of Tianjin Medical University, 300070 Tianjin, People’s Republic of China

**Keywords:** Oncogenes, Tumour biomarkers

## Abstract

Thyroid cancer (TC) is the most common endocrine tumor, amongst which anaplastic thyroid carcinoma (ATC) is the most deadly. Aurora-A usually functions as oncogenes, and its inhibitor Alisertib exerts a powerful antitumor effect in various tumors. However, the mechanism of Aurora-A in regulating TC cell energy supply remains unclear. In the present study, we demonstrated the antitumor effect of Alisertib and an association between high Aurora-A expression and shorter survival. Multi-omics data and in vitro validation data suggested that Aurora-A induced PFKFB3-mediated glycolysis to increase ATP supply, which significantly upregulated the phosphorylation of ERK and AKT. Furthermore, the combination of Alisertib and Sorafenib had a synergistic effect, further confirmed in xenograft models and in vitro. Collectively, our study provides compelling evidence of the prognostic value of Aurora-A expression and suggests that Aurora-A upregulates PFKFB3-mediated glycolysis to enhance ATP supply and promote TC progression. Combining Alisertib with Sorafenib has huge prospects for application in treating advanced thyroid carcinoma.

## Introduction

Thyroid cancer (TC) is the most common endocrine tumor worldwide and can be divided into differentiated thyroid cancer (DTC), anaplastic thyroid carcinoma (ATC), and medullary thyroid carcinoma (MTC) [1]. Differentiated thyroid cancer (DTC), including papillary thyroid carcinoma (PTC) and follicular thyroid carcinoma (FTC), account for 95% of all TC cases. After conventional treatment, a small portion of patients (5–10%) experience disease recurrence and progression. Multimodal therapy, including surgery, external beam radiation, watchful waiting and experimental trials, is indicated for advanced and treatment-refractory diseases and is associated with marginal survival benefits [2]. Although ATC and DTC are of the same origin, they exhibit different differentiation degrees and biological behaviors. Current evidence suggests albeit ATC accounts for only 1–2% of thyroid cancer cases, it is associated with 15–39% of thyroid cancer-related deaths. ATC patients have a 1-year overall survival of 20% and a historical median survival of about 5 months [3]. Understanding the underlying molecular mechanisms and identifying novel drug targets of advanced DTC and ATC are essential to developing novel prognostic evaluation and therapy strategies.

In mammals, the Aurora serine/threonine kinase family includes Aurora-A, Aurora-B, and Aurora-C. It is well-established that Aurora-A and Aurora-B regulate the cell cycle from G2 to cytokinesis and play prominent roles in most cell types [4]. Moreover, Aurora-A is required for many important mitotic events, including centrosome maturation, mitotic entry, mitotic spindle formation, and cytokinesis [5].

Overexpression or amplification of Aurora-A has been reported in many tumors, affecting various biological behaviors of tumor cells, such as activating cell cycle progression, promoting cell survival, inducing anti-apoptotic signaling, maintaining genomic instability, developing epithelial-mesenchymal transition (EMT), and maintaining cancer stemness [6, 7]. It acts as an oncogene and promotes tumorigenesis, suggesting it is a potential target for cancer treatment. Interestingly, several Aurora-A small-molecule inhibitors have been developed in recent years with promising antitumor activity in various tumors, including thyroid cancer [8, 9]. Alisertib has demonstrated remarkable anticancer activities by selectively inhibiting Aurora-A in preclinical studies and is the most advanced drug associated with 32 clinical trials [8]. Our previous study screened a small molecule inhibitor library and revealed that Alisertib yielded a superior inhibitory effect on the proliferation of ATC cells [10]. Nonetheless, the role of Aurora-A in advanced DTC and ATC progression and the potential clinical significance of Alisertib remains unclear.

Metabolic reprogramming, characterized by changes in cellular metabolic patterns, is the central contributor to promoting cancer progression. It is widely thought that cancer-related metabolic reprogramming has profound implications for gene expression, cell differentiation, and the cancer microenvironment [11]. Even under normal oxygenation, cancer cells still prefer to convert glucose to pyruvate through glycolysis, a phenomenon termed the Warburg effect, representing an essential part of metabolic reprogramming. Various mechanisms mediate glycolysis at different levels. The first rate-limiting step of glycolysis is the conversion of fructose-6-phosphate (F6P) to fructose-1,6-bisphosphate (F1,6P2) by 6-phosphofructo-1-kinase (PFK-1), which is activated by intracellular fructose 2,6- bisphosphate (F2,6BP). F2,6BP enhances the binding of PFK-1 for F6P and overrides the suppression of PFK-1 by ATP, enabling glycolytic flux through the PFK-1 checkpoint to F1,6P2 synthesis. The concentration of F2,6BP is controlled by phosphofructokinase 2/fructose-2,6-bisphosphatase (PFKFB). Though the four isozymes of PFKFB (PFKFB1–4) have high sequence homology (85%) of their core catalytic domains, they display distinct properties. Among them, PFKFB3 has the highest kinase/phosphatase ratio (710 times) and unique properties, being responsible for producing F2 and 6BP and promoting glycolytic flux [12]. PFKFB3 has been reported to play an important role in promoting tumor progression. An increasing body of evidence suggests that inhibition of PFKFB3 by chemical inhibitors or genetic attenuation significantly reduced glycolytic flux, ras-based transformation, and tumor growth in athymic mice [13–15]. In addition, inhibition of PFKFB3 has been reported to disrupt pathological angiogenesis and induce normalization of tumor vasculature, thereby reducing metastasis and improving chemotherapy efficacy [16]. However, it remains unclear what role PFKFB3 plays in thyroid cancer.

Sorafenib is a multitarget TKI that inhibits VEGFR2, VEGFR3, KIT kinases, PDGFR, RET/PTC, and the MAPK pathway. Owing to the promising preclinical and clinical results, Sorafenib was approved by US Food and Drug Administration (FDA) to treat ^131^I-refractory DTC in 2005 [17, 18]. However, Sorafenib failed to induce a sustainable response in DTC, leading to drug resistance [19]. In addition, preclinical studies suggested that Sorafenib has antitumor activity in ATC, and several drugs may exert a synergic effect with Sorafenib in reducing ATC cell growth. However, several clinical trials did not report favorable outcomes, suggesting that Sorafenib has a limited role in ATC [19]. Further studies are urgently needed to develop an appropriate Sorafenib-based combination strategy for aggressive TCs based on molecular characteristics.

This study aimed to investigate the therapeutic potential of Alisertib and its combination with Sorafenib in thyroid cancer. Moreover, we analyzed factors that determined thyroid cancer cell response to Alisertib and elucidated the effects of Aurora-A mediated PFKFB3 phosphorylation on glycolysis and biological behavior in thyroid cancer cells.

## Materials and methods

### Clinical data and tissue samples

This study was approved by the Tianjin Medical University Cancer Institute and the hospital ethics committee. Informed consent was obtained from each donor. 20 paracancerous thyroid samples, 126 DTC and 29 ATC samples were selected for immunohistochemistry (IHC) assays.

### IHC

Sections from human thyroid cancer (DTC and ATC), paracancerous thyroid tissue and xenograft tumors from mice were stained with antibodies to p-Aurora-A (host species: Rat, 44-1210 G Invitrogen), Aurora-A (host species: Rat, #ab52973, Abcam), Ki-67(host species: Rat,9027, CST) for IHC, according to a previously described protocol. Signals were visualized with a DAB substrate kit (Maixin Bio, China). Two experienced pathologists blinded to the patient information evaluated staining results. Aurora-A and p-Aurora-A expression levels were evaluated using the following criteria: The staining intensity was scored as 0 (negative), 1 (weak), 2 (moderate) and 3 (strong) points, while the positive cell count/total cell count (staining ratio) was evaluated with ImageJ software. The immunohistochemical score was obtained by the product of the staining intensity with the staining ratio; scores ≥150 were considered high expression. Ki-67 expression was quantified by the staining ratio. Hematoxylin and eosin staining was performed as previously described [2].

### Cell line and cell culture

In this study, we used 1 normal thyroid follicular epithelial cell (Nthy-ori3-1), 3 DTC cell lines (K-1, KTC-1, TPC-1) and 3 anaplastic thyroid cancer cell lines (8305 C, 8505 C and CAL-62) that were purchased from the American Type Culture Collection. All cell lines were identified by STR analysis, cultured at 37 °C and 5% CO_2_ in RPMI-1640 or DMEM (Gibco) supplemented with 10% fetal bovine serum, penicillin/streptomycin (5000 units/mL; Gibco) and l-glutamine (2 mM; Gibco). Alisertib (Selleck), ATP (Sigma), 2-deoxy glucose (2-DG, Selleck) and Sorafenib (Selleck) were used in this study.

### Western blotting

Cells were lysed with RIPA lysis buffer (Beyotime), and protein concentrations were determined by BCA. The samples were separated by SDS-PAGE. Gel proteins were transferred to PVDF membranes (Millipore), sequentially blocked with 5% nonfat milk (Solarbio), and then incubated with primary antibodies overnight at 4 °C. PVDF membranes were further incubated with secondary antibodies, and chemiluminescent detection was performed using the Western Blotting Detection Kit ECL (Human IgG) (Solarbio). Primary antibodies used for western blot included p-Aurora-A (host species: Rat, #3079, CST), Aurora-A (host species: Rat, #ab52973, Abcam), GAPDH (host species: Rabbit, #ab181602, Abcam), PFKFB3 (host species: Rat, # ab181861, Abcam), p-PFKFB3 (host species: Rat, # ab202291, Abcam), p-ERK (host species: Rat, #4370, CST), ERK (host species: Rat, #4695, CST), p-AKT (host species: Rat, #a4060, CST), and AKT (host species: Rat, #4685, CST). GST (host species: Rat, # ab252882, Abcam). ImageJ software was performed to quantify the band intensities of Western blot figures.

### Cell transfection and lentivirus infection

Small Interfering RNAs (siRNAs) were synthesized by GenePharma, Shanghai, China. siRNAs were transfected into cells using Lipofectamine 2000 (Invitrogen, Shanghai, China) according to the manufacturer’s instructions. After 48 h, the cells were collected for subsequent experiments. siRNAs were transfected into cells using Lipofectamine 3000 (Invitrogen) according to the manufacturer’s instructions. After 2 days, cells were collected for subsequent experiments. The human Aurora-A sequences were cloned into the PCDH vector (Addgene) and delivered by lentiviral infection. shRNA sequences were cloned into the PLKO.1 vector (Addgene) and delivered by lentiviral infection with lentiviruses generated by transfection of 293 T cells. The sequences of siRNA and shRNA are listed in Table 1 and Table 2

**Table 1:** The shRNA targeting sequence.

	Forward	Reverse
Aurora-A	CACATACCAAGAGACCTACAA	TTGTAGGTCTCTTGGTATGTG

**Table 2:** The siRNA sequences.

	Forward	Reverse
siPFKFB3-1	AGUUGUAGGAGCUGUACUG	CAGUACAGCUCCUACAACU
siPFKFB3-2	CGGGUGCAUGAUUGUGCUUAA	UUAAGCACAAUCAUGCACCCG

### RT-PCR

RT-PCR assays were performed as previously described [2]. Briefly, total RNA was extracted from cells using an RNeasy Mini Kit (Qiagen). Transcriptor First Strand cDNA Synthesis Kit (Takara) was performed to complete cDNA synthesis. FastStart Universal SYBR Green Master Mix (Takara) was used during PCR on an ABI ViiA7 system. The primers used are listed in Table 3.

**Table 3:** The primers of RT-PCR assays.

	Forward	Reverse
Aurora-A	GCAACCAGTGTACCTCATCCTG	AAGTCTTCCAAAGCCCACTGCC
Actin	CACCATTGGCAATGAGCGGTTC	AGGTCTTTGCGGATGTCCACGT

### Cell viability assay

A total of 1000–1500 cells were seeded per well in a 96-well plate. Then the viability of the cells was detected by Cell Counting Kit-8 (Cell Counting Kit-8, CCK-8) according to the manufacturer’s instructions.

### Transwell assay

Cell migration assays were performed using Transwell cells with 24-well polycarbonate membranes (8 mm pore size; Corning, USA). RPMI 1640 medium (500 µl) containing 10% fetal bovine serum was added to the lower chamber. 150 µl of cell suspension (1 × 10^6^ cells/ml) without serum was collected and incubated in the upper chamber at 37 °C, 5% CO_2_ for 24 h. Transwell cell chambers were fixed with 5% glutaraldehyde and stained with 0.1% crystal violet. For the cell invasion assay, the Transwell chamber was coated with Matrigel (BD Biosciences) before adding the cell suspension.

### ADP/ATP ratio

ADP/ATP ratios were measured using a commercial ADP/ATP kit obtained from Sigma-Aldrich (Darmstadt, Germany) according to the manufacturer’s instructions. Briefly, cells were incubated with ATP reagent for 1 min, and the luminescence was read for ATP detection. Cells were incubated with luminescence as background for 10 min prior to ADP measurements. Finally, the ADP reagent was added and incubated for 1 min. The luminescence was read, and ADP/ATP ratios were calculated according to the manufacturer’s instructions.

### Multi-omics analysis

Phosphoproteome and proteome analyses and RNA-seq were performed as previously described [20, 21]. After the adjudgment of proteome data, phosphoproteome data was used to compare the phosphorylation of protein in Aurora-A knockdown TPC-1 cells with the control group. Proteome analysis combined with RNA-seq was applied to compare the protein expression profile in TPC-1 cells with Aurora-A knockdown and the control group. To analyze The Cancer Genome Atlas (TCGA) data, we downloaded TCGA database data and divided the patients into two groups according to the expression level of Aurora-A. GSEA analysis was utilized in both groups to identify significantly enriched pathways in the Aurora-A upregulation group. Sequencing data were deposited in the Dryad database (https://datadryad.org/stash/share/FRYADEXXTfPZWs82hOEl_iJXNkn6_3uEQrpTs6hNU00).

### Immunoprecipitation (IP)

Immunoprecipitation was performed as previously described [22]. Briefly, cells were lysed with IP lysis buffer (Thermo Scientific, USA) containing the cocktail. Cell lysates were incubated with anti-Aurora-A antibody or PFKFB3 overnight at 4 °C, followed by immunoprecipitation with protein A + G agarose beads (Thermo Scientific, USA) for 2 h at 4 °C with shaking and washed 3 times with IP lysis buffer. The immunoprecipitate was eluted and analyzed by western blotting.

### Glutathione S-transferase (GST) pull-down

His-tagged PFKFB3 (Abcam, ab268355) was mixed with GST or GST-tagged Aurora-A (MCE, HY-P71199) were in binding buffer at 4 °C overnight, followed by the addition of glutathione-sepharose beads. After 2 h of incubation, the beads were washed five times with PBS and eluted with loading buffer, and the samples were examined by Western blotting.

### In vitro kinase assay

Substrate PFKFB3 (250 ng) was incubated with active Aurora-A (indicated dose) in a 30 μL reaction system (2ul 10X kinase assay buffer, 0.5 mM ATP) at 37 °C for 2 h. The reactions were stopped by adding 5× SDS loading buffer. Finally, the phosphorylation signal was detected by Western blotting.

### Seahorse XF Extracellular Flux analysis

The Seahorse XF Glycolysis Stress Test Kit (103020-100, Agilent Technologies) was used to measure the extracellular acidification rate (ECAR) based on the manufacturer’s instructions. Briefly, 2 × 104 cells were seeded per well in the XFe24 cell culture plate (102342-100, Agilent Technologies) and incubated at 37 °C overnight. Next day, medium was changed to Seahorse XF DMEM Medium(103575-100, Agilent Technologies) with 1 mM glutamine(103579-100, Agilent Technologies) and then cells were incubated at 37 °C for 60 min in the CO2 free incubator to balance the media pH and temperature. The extracellular acidification rate (ECAR) were monitored in baseline conditions and treated with 10 mM glucose, 1 µM oligomycin, 50 mM 2-DG. Data were normalized by the protein quantification and repeated in three times.

### Animal studies

The animal experiments were performed according to the IACUC protocol and approved by the Tianjin Medical University Cancer Institute and the Hospital Animal Care and Use Committee. The mice were allocated to different groups according to the random table method.

To construct the tumor xenograft model, NSG female mice (4 weeks old) were injected subcutaneously at 100 μl PBS with 1 × 105 CAL-62 or 8305 C cells to generate the xenograft model. When the average tumor volume reached 15 mm3, mice were randomly divided into a control group and several experimental groups. To evaluate the effect of Alisertib, mice were fed 2% hydroxy methylcellulose (HMC) or 1% HMC + 30 mg/kg of Alisertib daily. To confirm the synergistic effect of Alisertib and Sorafenib in vivo, mice were treated with 2% HMC, 1% HMC with 30 mg/kg Alisertib, 1% HMC with 30 mg/kg Sorafenib, and both Sorafenib and Alisertib, daily. Tumor growth was measured and recorded before the treatment began. After 12 days of treatment, the mice were sacrificed. The tumors were excised and fixed with 4% paraformaldehyde for further study.

To establish lung metastasis animal model, the Aurora-A upregulated and control KTC-1 cell lines with luciferase label were injected into NSG female mice (five mice per group) via the tail vein at a concentration of 1 × 106 cells/mouse. Metastatic tumor size was respectively measured using live animal imaging system. After 5 weeks, the mice were euthanized, and the lungs and livers were removed and fixed with 4% paraformaldehyde for further study.

### Statistical analysis

The data are presented as the mean ± SD of three independent experiments. Statistical analysis was performed using GraphPad Prism Ver. 7.0 (CA, USA). T-tests and ANOVA were used to determine the statistical significance according to the assumptions of the tests. Survival curves were evaluated by Kaplan–Meier analysis. Statistical significance is indicated by *P* < 0.05. The sample size was chosen referring to the previous studies [22, 23].

## Results

### Alisertib inhibits the proliferation, migration, invasion and Aurora-A phosphorylation of ATC cells in vitro and vivo

**Fig. 1:**
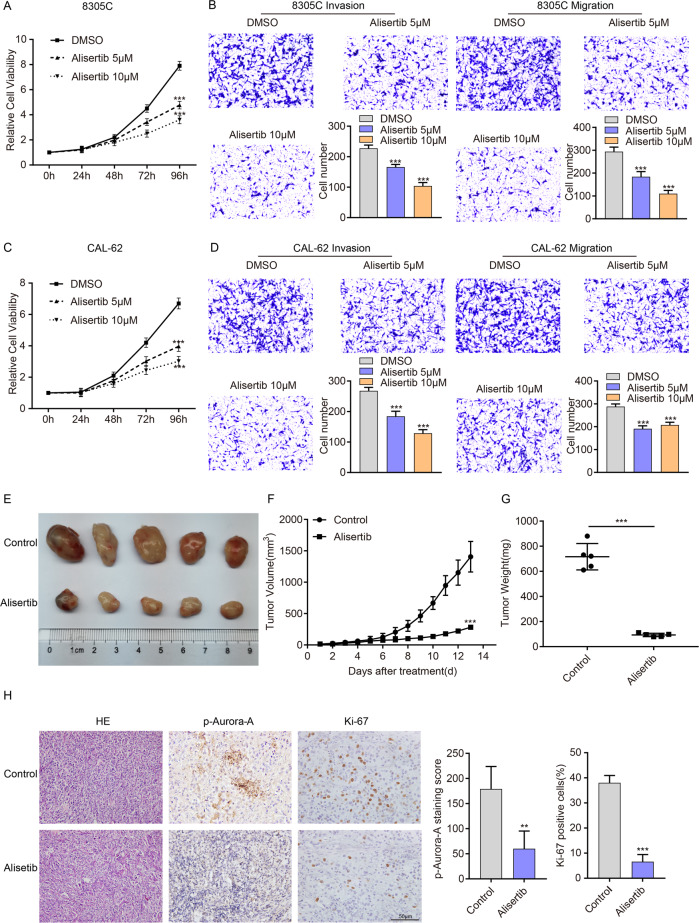
Alisertib inhibits the proliferation, migration, and invasion of ATC cells in vitro and vivo. **A**, **C** CCK-8 assays showed that Alisertib (5 μmol and 10 μmol) treatment significantly suppressed cell proliferation in 8305 C (**A**) and CAL-62 (**C**) cells in a dose-dependent pattern. **B**, **D** After treatment with the indicated concentration of Alisertib for 2 days, the migration/invasion assay was performed in 8305 C (**B**) and CAL-62 (**D**) cells. Representative images are shown. The above data are presented as the mean ± SD of three independent experiments. **E** Five representative tumors from NSG mice injected with CAL-62 are shown, *n* = 5 mice per group. The subcutaneous xenografts were dissected and are shown on day 13. **F**, **G** Tumor volumes were measured with growth curves (**F**) and tumor weights (**G**) in the control group (DMSO) and Alisertib group (30 mg/kg). **H** Representative IHC images and p-Aurora-A and Ki-67 staining in xenograft tumor specimens of control and Alisertib groups are shown. p-Aurora-A and Ki-67 expression were significantly decreased after treatment with Alisertib. Scale bars, 50 μm. The data are presented as the mean ± SD. All *P < 0.05, **P < 0.01, ***P < 0.001, compared with the control group.

Our previous studies indicated that Alisertib could suppress CAL-62 proliferation (Table 4) [10]. Therefore, we sought to further analyze the antitumor activity of Alisertib in thyroid tumors. As expected, Alisertib could effectively inhibit the proliferation, invasion, and migration of 8305 C and CAL-62 cells in a dose-dependent manner in vitro (Fig. 1A–D). We also detected the expression of Aurora-A and P-Aurora-A in ATC cells after Alisertib treatment in vitro and found that Alisertib could reduce the expression of p-Aurora-A (the active form of Aurora-A functioning as a kinase) in a dose-dependent manner, but had no significant effect on the expression of total Aurora-A (Supplementary Fig. 1A). This finding demonstrated that Alisertib exerted an inhibitory effect on Aurora-A kinase activity in thyroid cancer.

**Table 4:** Information of Alisertib in drug library.

Inhibitor	CAS No.	Target	Cell viability(%)
MLN8237(Alisertib)	1028486-01-2	Aurora Kinase inhibitor	43.37

The tumor xenograft model was established by subcutaneous injection of CAL-62 cells in NSG mice, and Alisertib was administered after tumor formation. Alisertib effectively inhibited tumor proliferation in vivo, with a reduction in tumor size (Fig. 1E, F) and weight (Fig. 1G). Immunohistochemical staining showed that Ki67 and P-Aurora-A expression in the treatment group was significantly lower than in the control group (Fig. 1H).

This finding indicated that Alisertib exerts a potent inhibitory effect on Aurora-A activity, proliferation and migration in thyroid cancer cells in vivo and in vitro.

### Aurora-A promotes the proliferation, migration and invasion of thyroid cancer cells, and its elevation is correlated to a poor prognosis in TC patients

**Fig. 2:**
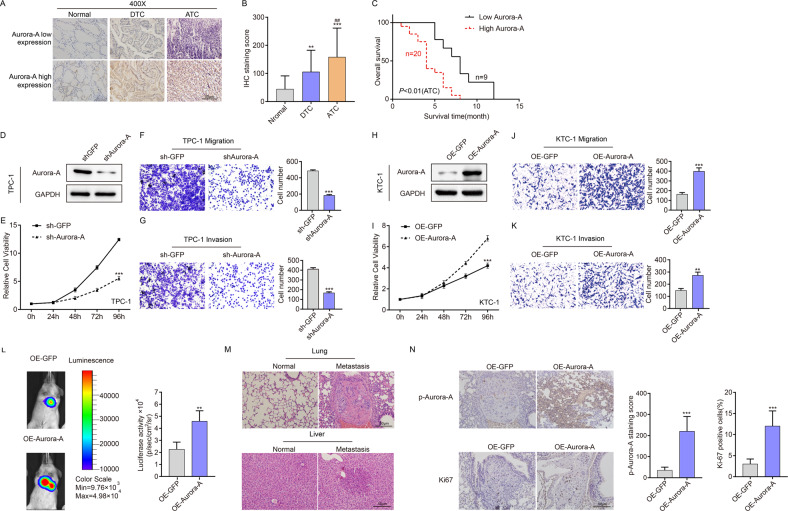
Aurora-A promotes the proliferation, migration and invasion of TC cells, and its expression is correlated to the poor prognosis of TC. **A** Representative IHC images of low expression and high expression of Aurora-A were shown. Scare bar is 50 µm. **B** Varying expression levels of Aurora-A in 20 normal samples, 126 DTC samples and 29 ATC samples. **P < 0.01, ***P < 0.001, compared with control normal samples. ^#^*P* < 0.05, ^##^*P* < 0.01, ^###^*P* < 0.001, compared with DTC samples. **C** Kaplan–Meier survival analysis showed that high Aurora-A expression correlated with short survival time in ATC patients. **D** Aurora-A expression in TPC-1 cells was detected by western blotting in the Aurora-A knockdown group. **E** CCK-8 assays showed that Aurora-A downregulation significantly enhanced cell proliferation in TPC-1 cells. *P < 0.05, **P < 0.01, ***P < 0.001, compared with control group. **F**, **G** Representative images of transwell assay of migration (**F**) and invasion (**G**) of TPC-1 cells with or without Aurora-A knockdown. *P < 0.05, **P < 0.01, ***P < 0.001, compared with control group. **H** Aurora-A expression in KTC-1 cells was detected by western blotting in Aurora-A overexpressed group. **I** CCK-8 assays showed that Aurora-A upregulation significantly enhanced cell proliferation in KTC-1 cells. *P < 0.05, **P < 0.01, ***P < 0.001, compared with control group. **J**, **K** Representative images of transwell assay of migration (**J**) and invasion (**K**) of KTC-1 cells with or without Aurora-A overexpression. *P < 0.05, **P < 0.01, ***P < 0.001, compared with control group. Above data are presented as the mean ± SD of three independent experiments. **L** Representative bioluminescent images and statistical analysis of the two groups on days 35 after tail vein injection (*n* = 5 per group) **M** Representative H&E staining of metastatic tumors in the lungs and livers. **N** Representative immunohistochemical staining for Ki-67 and p-Aurora-A. ***P < 0.001.

To corroborate our pharmacological results, we investigated the clinical relevance of Aurora-A expression in TC patients. The mRNA expression of Aurora-A in several TC cell lines (including DTC and ATC) was detected, suggesting that the expression of Aurora-A in ATC cell lines was significantly higher than in DTC cell lines and normal thyroid cell lines (Supplementary Fig. 1B). Then, we performed immunohistochemical staining for Aurora-A in DTC and ATC sections to understand the relationship between Aurora-A expression and clinicopathological features of TC. Higher Aurora-A expression was observed in ATC than in DTC and was associated with a poor prognosis in ATC patients (Fig. 2A–C).

To further verify the biological function of Aurora-A in TC, we overexpressed Aurora-A in KTC-1 cell lines and found that Aurora-A overexpression significantly enhanced KTC-1 cell proliferation, migration, and invasion ability (Fig. 2D–G). Consistently, Aurora-A downregulation significantly reduced TPC-1 cell viability, migration and invasion. Likewise, Aurora-A overexpression increased tumor metastasis in tail vein injection model (Fig. 2L, M). IHC staining of metastatic tumor tissue sections revealed increased p-Aurora-A and Ki-67 staining (Fig. 2N). The above findings suggest that Aurora-A is a promising target in TC cells, and increased Aurora-A expression could be a potential marker of poor outcomes in TC patients.

### Multiomics and bioinformatics analyses suggest Aurora-A regulates the glycolysis pathway in TC

**Fig. 3:**
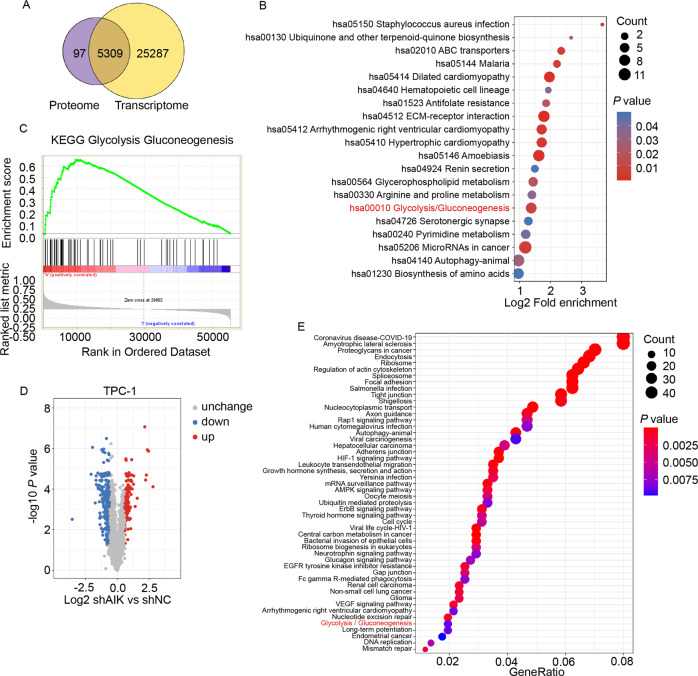
Multiomics and bioinformatics analyses indicated Aurora-A regulates the glycolysis in TC cells. **A** The Venn plot of proteome and RNA-seq revealed 5309 genes had significant alterations at the mRNA and protein levels after Aurora-A knockdown. **B** KEGG analysis of 5309 genes showed significant alterations in both proteome and RNA-seq. Glycolysis pathway can be remarkably enriched. **C** Analysis of TCGA database revealed Aurora-A was positively correlated with the glycolysis pathway. **D** Volcano plot highlighting phosphorylation changes of proteins. 318 proteins with 423 sites were identified with greater than 1.5-fold change in Aurora-A knockdown relative to control TPC-1 cells and plotted in red (upregulation) or blue (downregulation), respectively. **E** KEGG analysis of the phosphorylation-changed proteins after Aurora-A knockdown in TPC-1 cells. The glycolysis pathway was significantly enriched.

To further explore the mechanism underlying the stimulatory effect of Aurora-A on TC progression, transcriptome analysis combined with protein profiling was performed, revealing significant enrichment of the glycolysis pathway (Fig. 3A–B). For further confirmation, thyroid cancer data from TCGA database was obtained. GSEA analysis was performed according to Aurora-A expression. Glycolysis and gluconeogenesis pathway was also enriched in the Aurora-A high expression group (Fig. 3C). In addition, phosphoproteome profiling was performed on the Aurora-A-downregulated TPC-1 and the control group. 318 proteins with 423 sites showed significant changes in phosphorylation level (Fig. 3D). KEGG analysis revealed that multiple pathways, including the glycolysis pathway, were significantly enriched (Fig. 3E). PFKFB3 was further identified, and the phosphorylation level of the S461 site was significantly altered (Table 5). The above data suggested that Aurora-A may promote TC progression via glycolysis, and PFKFB3 may be the candidate substrate.

**Table 5:** PFKFB3 was identified by phosphoproteome and the phosphorylation level at S461 was significantly altered.

Protein accession	Q16875
Position	461
Amino acid	S
Protein description	6-phosphofructo-2-kinase / fructose-2,6-bisphosphatase
Gene name	PFKFB3
Localization probability	0.994515
TPC-1 shAurora-A/control Ratio	0.431
TPC-1 shAurora-A/control *P* value	13889999997507E-06

### Aurora-A interacts with PFKFB3 and mediates PFKFB3 phosphorylation to regulate glycolysis in TC cells

**Fig. 4:**
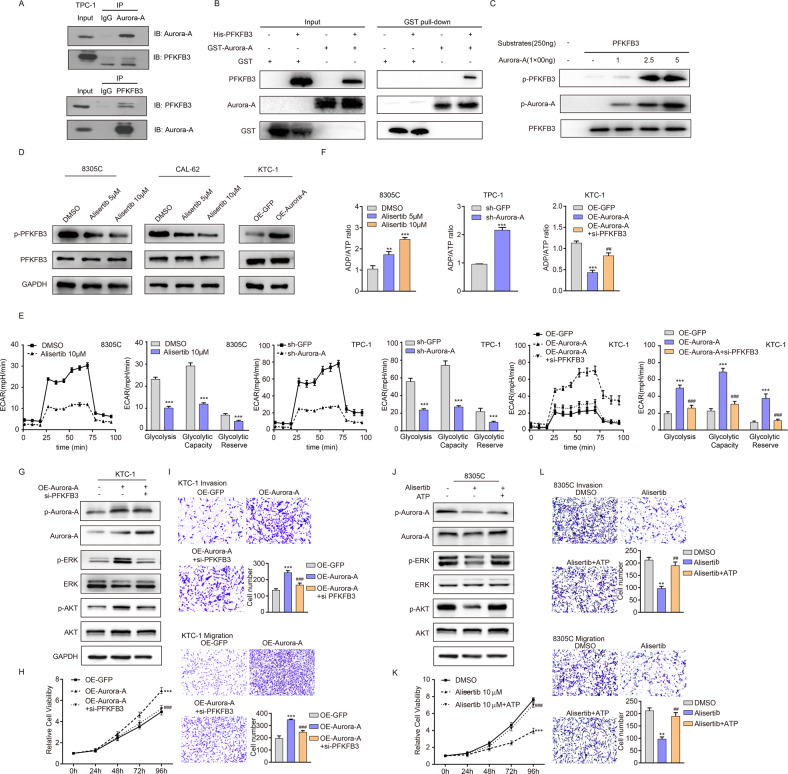
Aurora-A-mediated PFKFB3 phosphorylation regulates the glycolysis of TC cells. **A** The interaction between Aurora-A and PFKFB3 was verified by an IP assay in TPC-1 cells. **B** Interaction between Aurora-A and PFKFB3 is examined by GST pull-down. **C** Phosphorylation level on PFKFB3 was determined by Anti-PFKFB3 (phospho S461) antibody after in vitro kinase assay. **D** The expression of p-PFKFB3/PFKFB3 in KTC-1 cells with Aurora-A overexpression and Alisertib-treated 8305 C and CAL-62 cells was analyzed by western blotting. **E** Comparable level of glycolysis, glycolytic reserve and capacity of 8305 C, TPC-1, and KTC-1 cells measured by Seahorse XF Extracellular Flux analysis. ECAR = extracellular acidification rate. **F** The ADP/ATP ratio was measured in KTC-1 cells with Aurora-A overexpression or combined with siRNA targeting PFKFB3, in TPC-1 cells with Aurora-A knockdown and in 8305 C cells with Alisertib treatment, respectively. **G** The expression of p-Aurora-A, Aurora-A, p-ERK, ERK, p-AKT, AKT in KTC-1 cells with Aurora-A overexpression or combined with siRNA targeting PFKFB3. **H**, **I** The proliferation, migration and invasion ability in KTC-1 cells with Aurora-A overexpression or combined with siRNA targeting PFKFB3 was analyzed by CCK-8 assay (**H**) and transwell of migration and invasion assay (**I**), respectively. *P < 0.05, **P < 0.01, ***P < 0.001, compared with control group. ^#^*P* < 0.05, ^##^*P* < 0.01, ^###^*P* < 0.001, compared with Aurora-A upregulation group. **J** Western blotting analyzed the expression of p-Aurora-A, Aurora-A, p-ERK, ERK, p-AKT, and AKT in 8305 C cells with Alisertib treatment or combined with exogenous ATP (2 mM). **K** CCK-8 assays showed that Alisertib (10 μmol) significantly inhibited cell proliferation in 8305 C, which can be remarkably restored by exogenous ATP (2 mM) supplementation. *P < 0.05, **P < 0.01, ***P < 0.001, compared with control group. ^#^*P* < 0.05, ^##^*P* < 0.01, ^###^*P* < 0.001, compared with the Alisertib-treated group. **L** Transwell of migration and invasion assay detected the migration and invasion ability of 8305 C cells pretreated with Alisertib for 2 days or combined with exogenous ATP (2 mM). *P < 0.05, **P < 0.01, ***P < 0.001, compared with control group. ^#^*P* < 0.05, ^##^*P* < 0.01, ^###^*P* < 0.001, compared with the Alisertib-treated group. The data are presented as the mean ± SD of three independent experiments.

To our knowledge, the relevance of Aurora-A with PFKFB3 is not defined, especially in thyroid cancer. To validated the interaction between Aurora-A and PFKFB3, IP (Fig. 4A) and GST pull-down assay (Fig. 4B) was performed, revealing the physiological interaction between Aurora-A and PFKFB3. Based on the reported Aurora-A function and obtained multiomic data, we hypothesized that Aurora-A could directly phosphorylate PFKFB3 at S461 site. To this end, in vitro kinase assay was performed, revealing that recombinant Aurora-A could consistently phosphorylate recombinant PFKFB3 at S461 (Fig. 4C). Then we detected the expression changes of p-PFKFB3 and total PFKFB3 after treatment with Alisertib or knockdown Aurora-A, and found that Aurora-A activity blockage could reduce the level of p-PFKFB3 but had no effect on the level of total PFKFB3 (Fig. 4D, Supplementary Fig. 2 and Supplementary Fig. 3A). Consistently, Aurora-A overexpression in KTC-1 cell lines induced PFKFB3 phosphorylation without altering the total PFKFB3 expression level (Fig. 4D and Supplementary Fig. 3A). Above data coherently demonstrate that as a bona fide substrate of Aurora-A, PFKFB3, is phosphorylated at S461.

Overwhelming evidence substantiates that increased expression or phosphorylation of PFKFB3 facilitates the progression and glycolysis in various cancers [24, 25]. To confirm the tumor-promoting function of PFKFB3 in TC, we used two different siRNAs to knock down PFKFB3 in KTC-1 and found that PFKFB3 knockdown significantly increased the ADP/ATP ratio and reduced the phosphorylation level of ERK and AKT (Supplementary Fig. 4A, B). These results indicated that PFKFB3 induced the activation of MAPK and PI3K-AKT signaling pathways by enhancing the energy supply in TC cells. To demonstrate whether Aurora-A promote TC progression via PFKFB3, we performed Seahorse XF Extracellular Flux assay. Decreased Aurora-A activity or expression significantly attenuated the EACR, glycolytic capacity, and glycolytic reserve. Consistently, Aurora-A upregulation enhanced the glycolysis in KTC-1, which was interrupted by PFKFB3 knockdown (Fig. 4E). The glycolysis inhibitor 2-DG reserved the cell proliferation, migration and invasion in Aurora-A-overexpressed KTC-1 cells, also suggesting that Aurora-A promoted tumor progression by mediating PFKFB3-mediated glycolysis (Supplementary Fig. 5A–C). To validate that Aurora-A-regulated PFKFB3 phosphorylation played a carcinogenic role by affecting the energy supply of TC cells, we detected the ADP/ATP ratio after altering Aurora-A activity and found that Aurora-A activity was significantly negatively correlated with ADP/ATP ratio (Fig. 4F).

Next, we determined the role of Aurora-A and ATP supply in maintaining MAPK and PI3K-AKT signaling pathways in TC cells. As shown in Fig. 4G, H and Supplementary Fig. 3B, PFKFB3 downregulation could reverse the activation of the MAPK pathway and PI3K-AKT pathway and the proliferation, migration and invasion ability in Aurora-A-overexpressed KTC-1 cells. In addition, treatment with Alisertib significantly decreased the phosphorylation of ERK and AKT, while exogenous ATP mitigated the repression effect. The suppressed cell proliferation, migration and invasion were also reversed by supplementation of ATP (Fig. 4J–L and Supplementary Fig. 3C). The above results suggest that Aurora-A-mediated PFKFB3 phosphorylation affects the energy production of tumor cells by regulating glycolysis and thus plays a pro-carcinogenic role in tumor cells.

### Targeting Aurora-A improves the efficacy of Sorafenib treatment in TC cells

**Fig. 5:**
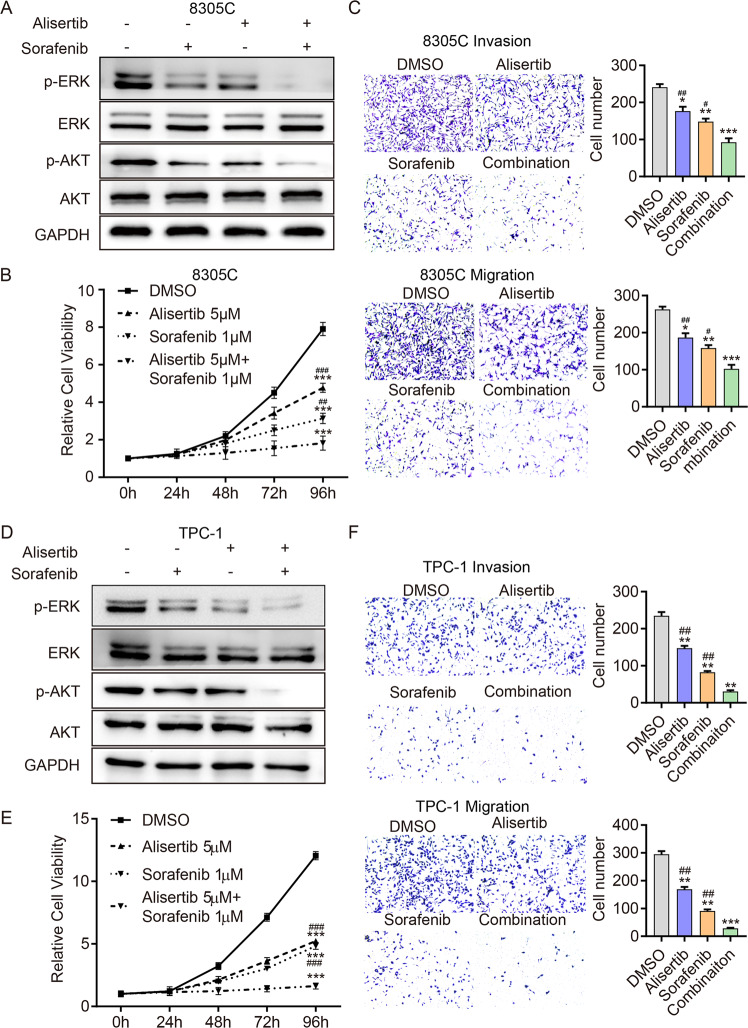
Targeting Aurora-A improves the efficacy of Sorafenib treatment in TC cells in vitro. **A**, **D** Western blotting detected p-ERK, ERK, p-AKT, and AKT expression in 8305 C (**A**) and TPC-1 (**D**) cells, respectively, exposed to Alisertib (5 μmol), Sorafenib (1 μmol) and their combination for 48 h. **B**, **E** The CCK-8 assay was performed in 8305 C (**B**) and TPC-1 (**E**) cells exposed to Alisertib (5 μmol), Sorafenib (1 μmol) and both drugs for 96 h. *P < 0.05, **P < 0.01, ***P < 0.001, compared with control group. ^#^*P* < 0.05, ^##^*P* < 0.01, ^###^*P* < 0.001, compared with the combination group. **C**, **F** Transwell of migration and invasion assay detected the migration and invasion ability of 8305 C (**C**) and TPC-1 (**F**) cells pretreated with Alisertib (5 μmol), Sorafenib (1μmol) or both drugs for 2 days. *P < 0.05, **P < 0.01, ***P < 0.001, compared with the control group. ^#^*P* < 0.05, ^##^*P* < 0.01, ^###^*P* < 0.001, compared with the combination group. The data are presented as the mean ± SD of three independent experiments.

To determine whether the combination of Alisertib and Sorafenib was more effective in TC cell lines, western blot, viability (CCK8) assay, and transwell assay were performed. As expected, compared with the monotherapy and control group, the combination strategy significantly inhibited the phosphorylation level of ERK AKT in both 8305c (Fig. 5A and Supplementary Fig. 3D) and TPC-1 cells (Fig. 5D and Supplementary Fig. 3E). Correspondingly, the proliferation (Fig. 5B, E), migration and invasion ability (Fig. 5C, F) of 8305c and TPC-1 cells were significantly suppressed in the combination group. These data suggest that the combination of Alisertib and Sorafenib could potently inhibit DTC cell proliferation in vitro.

**Fig. 6:**
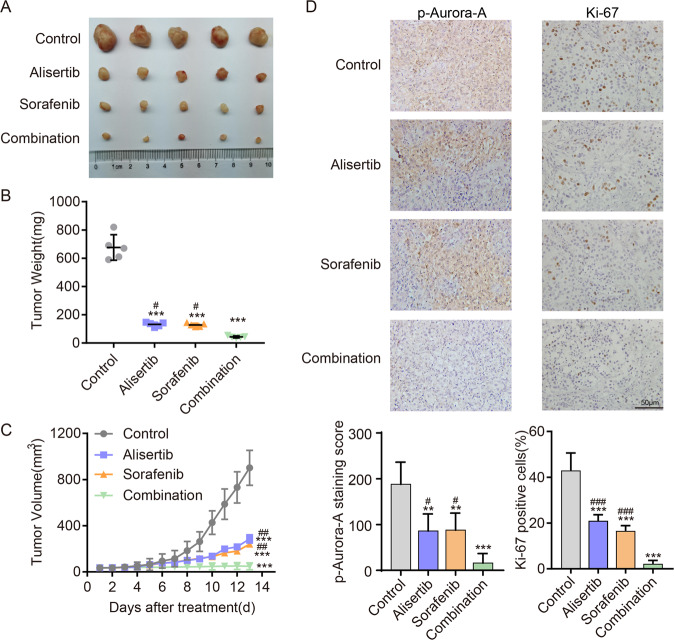
The combination of Alisertib and Sorafenib is a promising therapeutic approach for advanced TC in vivo. **A** Representative images of subcutaneous xenografts in NSG mice injected 8305 C cells treated with Alisertib (30 mg/kg), Sorafenib (30 mg/kg) or both drugs. *n* = 5 mice per group. The subcutaneous xenografts were dissected on day 13. **B**, **C** Tumor volumes were measured with tumor weights (**B**) and growth curves (**C**) in each group. **D** The expression levels of Ki67 and p-Aurora-A in xenografts of each group were assessed by immunohistochemical staining. *P < 0.05, **P < 0.01, ***P < 0.001, compared with control group. ^#^*P* < 0.05, ^##^*P* < 0.01, ^###^*P* < 0.001, compared with the combination group. The data are presented as the mean ± SD.

To validate our results, mice bearing 8305c xenografts were analyzed to examine the antitumor activity of the Alisertib/Sorafenib combination strategy. Compared with mice in the monotherapy group, mice in the combination group exhibited remarkably reduced tumor volumes and weight (*P* < 0.05, Fig. 6A–C). The expression of p-Aurora-A and Ki-67 in the combination group was significantly lower than in other groups (Fig. 6D). The effects of Alisertib or Sorafenib alone were similar to the above in vitro results. These results indicated that the Aurora-A inhibitor combined with Sorafenib could serve as a powerful therapeutic approach for TC, especially in cases where monotherapy has limited efficacy (Fig. 6).

## Discussion

In this study, we explored the expression pattern of Aurora-A in thyroid carcinoma and its relationship with patient prognosis. Importantly, we found that targeting Aurora-A by Alisertib can suppress the proliferation and epithelial-mesenchymal transformation of anaplastic thyroid cancer cells. Mechanistically, Aurora-A acts as a protein phosphorylation kinase that directly interact with and phosphorylate PFKFB3 at S461, in turn promoting the glycolysis process of tumor cells and causing metabolic reprogramming of tumor cells. More importantly, we substantiated that Alisertib significantly enhances the antitumor effect of Sorafenib, thus providing a new combination therapy strategy for thyroid cancer patients.

The abnormal upregulation of Aurora-A has been documented in tumors of the breast, ovary, prostate, cervix, lung, head, and neck and correlated with a poor prognosis. Our study yielded consistent results as the overexpression of Aurora-A correlated with a poor prognosis in TC patients, and Aurora-A expression in ATC was significantly higher than in DTC.

Multiomics experiments consistently enriched Glycolysis pathway after the alteration of Aurora-A activity, revealing a strong and broad effect of Aurora-A in cancer metabolic reprogramming, which was adequately confirmed by Seahorse analysis and 2-DG treatment. Studies revealed that glycolysis-related gene expression were in general linked with aggressiveness and poor prognosis in thyroid cancer [26]. In the in vitro experimental settings, PTC cell lines (BCPAP and TPC-1 cells) are more glycolytic than Nthy-ori 3-1 cells, as demonstrated by higher glucose uptake, LDH production and glycolysis-related gene expression. However, the detailed role and mechanism of glycolysis in thyroid cancer was still unclear. A few studies revealed that Aurora-A can induced glycolysis through activation of glycolysis-related enzymes [27] or enhanced glycolysis-related gene expression [28]. However, in thyroid cancer, the role of Aurora-A on glycolysis and its intrinsic mechanism have not been evaluated.

As a classical glycolysis-related gene, PFKFB3 was further picked up and S461 site phosphorylation was considered as potential target. S461 of PFKFB3 has been demonstrated as a key phosphorylation site for its stimulatory effect on F2,6BP production, lactate secretion, and proliferation in cancer cells [29]. Our work demonstrated PFKFB3 is a novel substrate of Aurora-A, and a major determinant for Aurora-A-induced cancer progression. To our knowledge, our study uncovered a novel mechanism of Aurora-A as an oncogene to promote tumor cell glycolysis and progression, especially in thyroid cancer. Taken together, our findings corroborated that Aurora-A affects the glycolytic flow of tumor cells through distinct mechanisms, and suggested that Aurora-A promoted glycolysis by increasing the phosphorylation level of the PFKFB3 S461 site in TC cells.

The advantages of accelerated glycolytic flux in tumors remain controversial [30, 31]. Our data suggested that PFKFB3-regulated glycolysis can promote the proliferation and invasion of TC cells by enhancing the energy supply, an important mechanism of Aurora-A-mediated TC cell progression. Sufficient ATP supplementation is necessary for protein phosphorylation to promote the activation of the cell signal transduction pathway [32]. Our study revealed that PFKFB3-mediated ATP supply contributed to the stimulatory effect of Aurora-A on the MAPK pathway and PI3K pathway, which are sensitive to ATP levels. The supplementation of exogenous ATP in PFKFB3 knockdown thyroid cancer cells can effectively restore the tumor suppressive effect.

Our research has proved that in thyroid cancer, PFKFB3 is the main substrate for Aurora-A to function as an oncogene. By activating glycolysis pathway, the aberrant expression of Aurora-A is of great significance for TC progression. Considering the specific and vital role of the MAPK and AKT pathways in TC progression, above finding also indicated that inhibition of Aurora-A activity only or combined with other targeted drugs has potential clinical value for advanced TC therapy.

Similar to our findings, Aurora-A inhibitors have showed noteworthy antitumor effect in various cancers [33, 34]. However, due to the genomic heterogeneity of cancer, these studies also rindicate that the use of Alisertib may have a potential weakness in terms of recurrence. Our study revealed a synergistic effect between Alisertib and Sorafenib in advanced DTC and ATC. We demonstrated the efficacy of this combination in thyroid cancer and identified a new mechanism, indicating Aurora-A inhibitors have huge prospects for application in advanced TC therapy either alone or in combination. The combination of PFKFB3 inhibitor and Sorafenib has been demonstrated to have a synergistic effect on the treatment of liver cancer, consistent with the present study findings. Considering the limited role of Sorafenib in ATC, the combined strategy may be of great significance in effectively inhibiting the proliferation of ATC cells.

A previous study found that Aurora-A binds to c-MYC in thyroid cancer, which partially explains the mechanism of its carcinogenic effect [9]. The present study provides hitherto undocumented evidence that Aurora-A phosphorylates PFKFB3 and mediates the regulation of glycolysis and metabolic reprogramming. We also found that targeting Aurora-A could effectively inhibit therapeutic resistance to Sorafenib caused by metabolic reprogramming. Our study provides novel insights into the mechanism by which Aurora-A exerts its carcinogenic effects.

In summary, our study uncovered the prognostic value of Aurora-A expression in thyroid cancer and a new carcinogenic mechanism: Aurora-A can increase PFKFB3-mediated glycolysis to activate the MAPK and AKT pathways. Interestingly, the combination of Aurora-A inhibitors and Sorafenib may be a promising treatment strategy for aggressive thyroid carcinoma.

## Supplementary information

aj-checklist

supplementary legends

Supplementary Fig.1

Supplementary Fig.2

Supplementary Fig.3

Supplementary Fig.4

Supplementary Fig.5

WB original data

## Data Availability

The authors declared that all the data and materials are available on reasonable request.
