# Supplementary material for: Targeting Aurora-A inhibits tumor progression and sensitizes thyroid carcinoma to Sorafenib by decreasing PFKFB3-mediated glycolysis
Source: Cell Death Dis. 2023 Mar 29;14(3):224. doi: 10.1038/s41419-023-05709-z (PMC10060208; doi:10.1038/s41419-023-05709-z)
Supplement: Supplementary file 2 — supplementary legends [file 41419_2023_5709_MOESM2_ESM.docx]

**Supplementary FIG1**

A. The expression of Aurora-A and p-Aurora-A in 8305C and CAL-62 cells with or without 10μmol Alisertib treatment were detected by western blotting.

B. Aurora-A expression in Nthy-ori-3-1, DTC and ATC cell lines were detected by real-time PCR.

**Supplementary FIG2**

A. The expression of p-PFKFB3/PFKFB3 in TPC-1 cells with Aurora-A downregulation was analyzed by western blotting.

**Supplementary FIG3**

Quantification of and the statistical analysis of Western blots in FIG4 and FIG5.

A. Analysis of the expression of p-PFKFB3/PFKFB3 in KTC-1 cells with Aurora-A overexpression and Alisertib-treated 8305C and CAL-62 cells. *P < 0.05, **P < 0.01, ***P < 0.001, compared with control group.

B. Analysis of the expression of p-Aurora-A/Aurora-A, p-ERK/ERK and p-AKT/AKT in KTC-1 cells with Aurora-A overexpression or combined with siRNA targeting PFKFB3. *P < 0.05, **P < 0.01, ***P < 0.001, compared with control group. #P < 0.05, ##P < 0.01, ###P < 0.001, compared with Aurora-A upregulation group.

C. Analysis of the expression of p-Aurora-A/Aurora-A, p-ERK/ERK and p-AKT/AKT in 8305C cells with Alisertib treatment or combined with exogenous ATP (2mM). *P < 0.05, **P < 0.01, ***P < 0.001, compared with control group. #P < 0.05, ##P < 0.01, ###P < 0.001, compared with the Alisertib-treated group.

D, E. Analysis of the expression of p-ERK/ERK and p-AKT/AKT in 8305C (D) and TPC-1 (E) cells, respectively, exposed to Alisertib (5μmol), Sorafenib (1μmol) and their combination for 48h.

**P* < 0.05, ***P* < 0.01, ****P* < 0.001, compared with control group. #*P* < 0.05, ##*P* < 0.01, ###*P* < 0.001, compared with the combination group.

The data are presented as the mean ± SD of three independent experiments

**Supplementary FIG4**

A. The ADP/ATP ratio in KTC-1 cells with or without siRNA targeting of PFKFB3.

B. The expression of PFKFB3, p-ERK, ERK, p-AKT and AKT in KTC-1 cells with or without siRNA targeting of PFKFB3 were detected by western blotting.

**Supplementary FIG5**

A. CCK-8 assays showed that Aurora-A overexpression significantly inhibited cell proliferation in KTC-1, which can be remarkably interrupted by exogenous 2-DG (10mM) supplementation.

B, C. Transwell of migration and invasion assay was performed in Aurora-A overexpressed KTC-1 or combined with 2-DG (10mM).

**P* < 0.05, ***P* < 0.01, ****P* < 0.001, compared with control group. #*P* < 0.05, ##*P* < 0.01, ###*P* < 0.001, compared with the Aurora-A upregulation group.

The data are presented as the mean ± SD of three independent experiments.

**Supplementary data**

WB original image
