# Supplementary figures and images for: Targeting Aurora-A inhibits tumor progression and sensitizes thyroid carcinoma to Sorafenib by decreasing PFKFB3-mediated glycolysis
Source: Cell Death Dis. 2023 Mar 29;14(3):224. doi: 10.1038/s41419-023-05709-z (PMC10060208; doi:10.1038/s41419-023-05709-z)

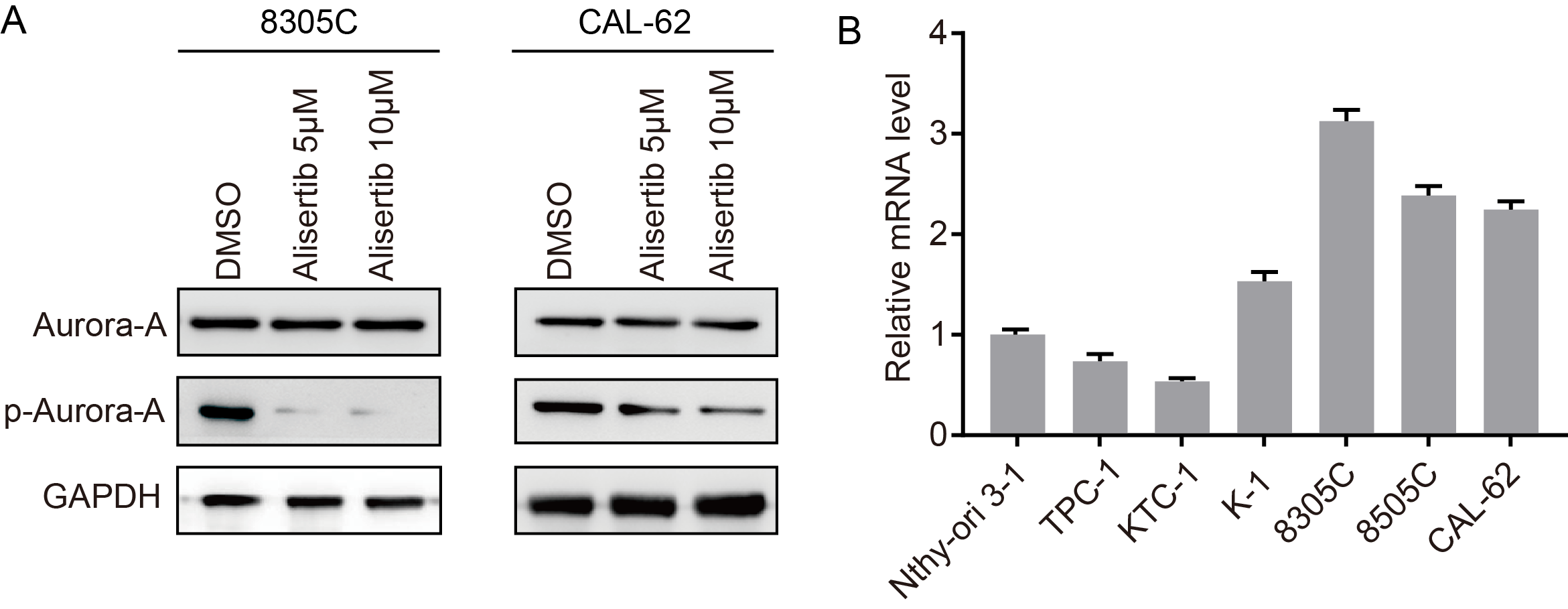

Supplement: Supplementary file 3 — Supplementary Fig.1 [file 41419_2023_5709_MOESM3_ESM.tif]

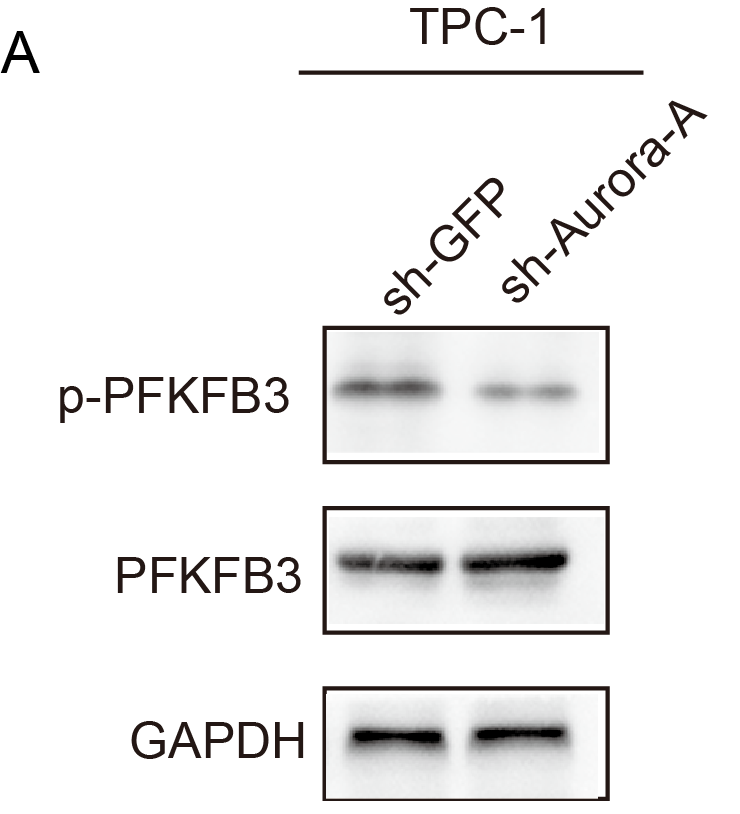

Supplement: Supplementary file 4 — Supplementary Fig.2 [file 41419_2023_5709_MOESM4_ESM.tif]

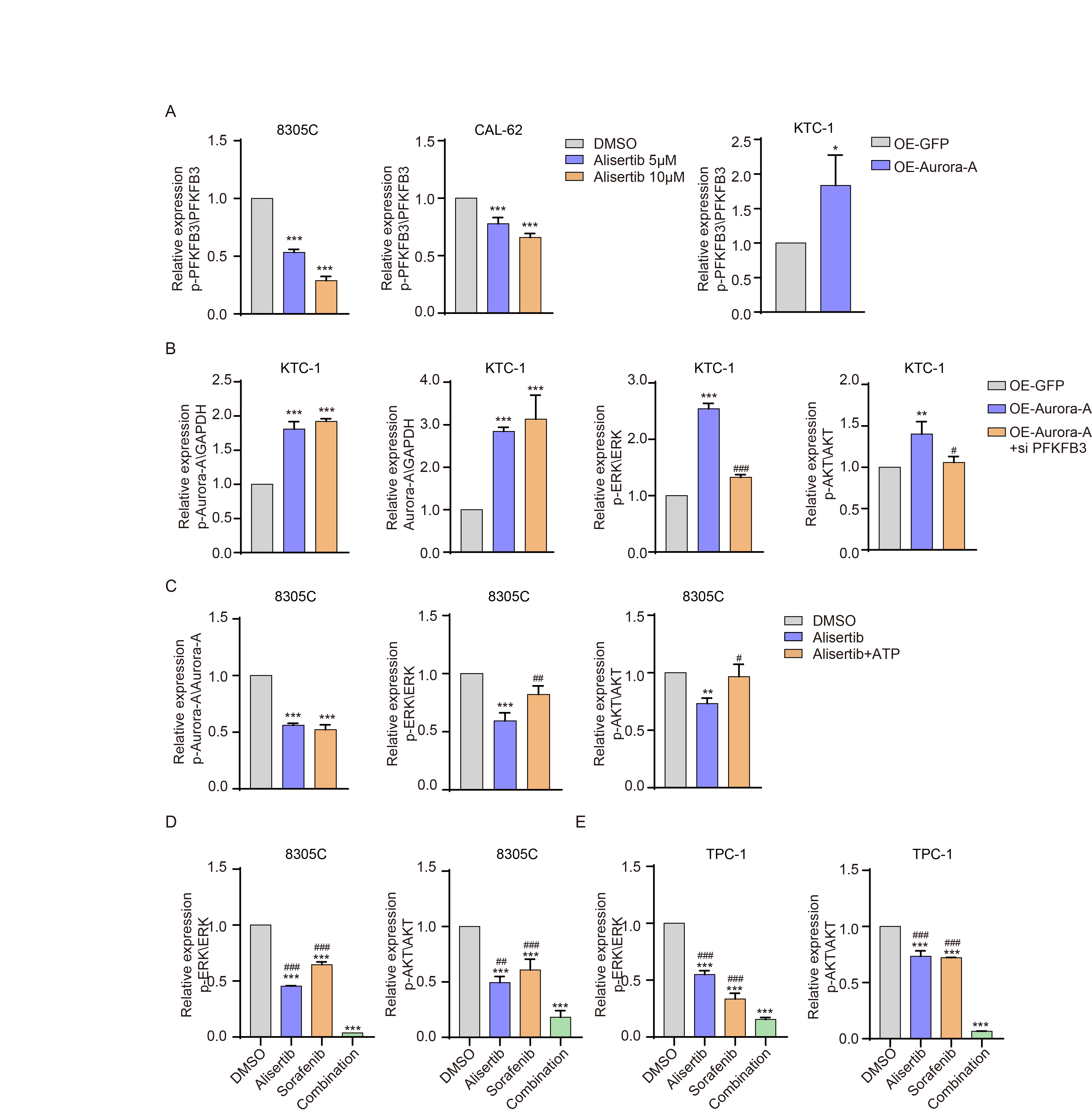

Supplement: Supplementary file 5 — Supplementary Fig.3 [file 41419_2023_5709_MOESM5_ESM.tif]

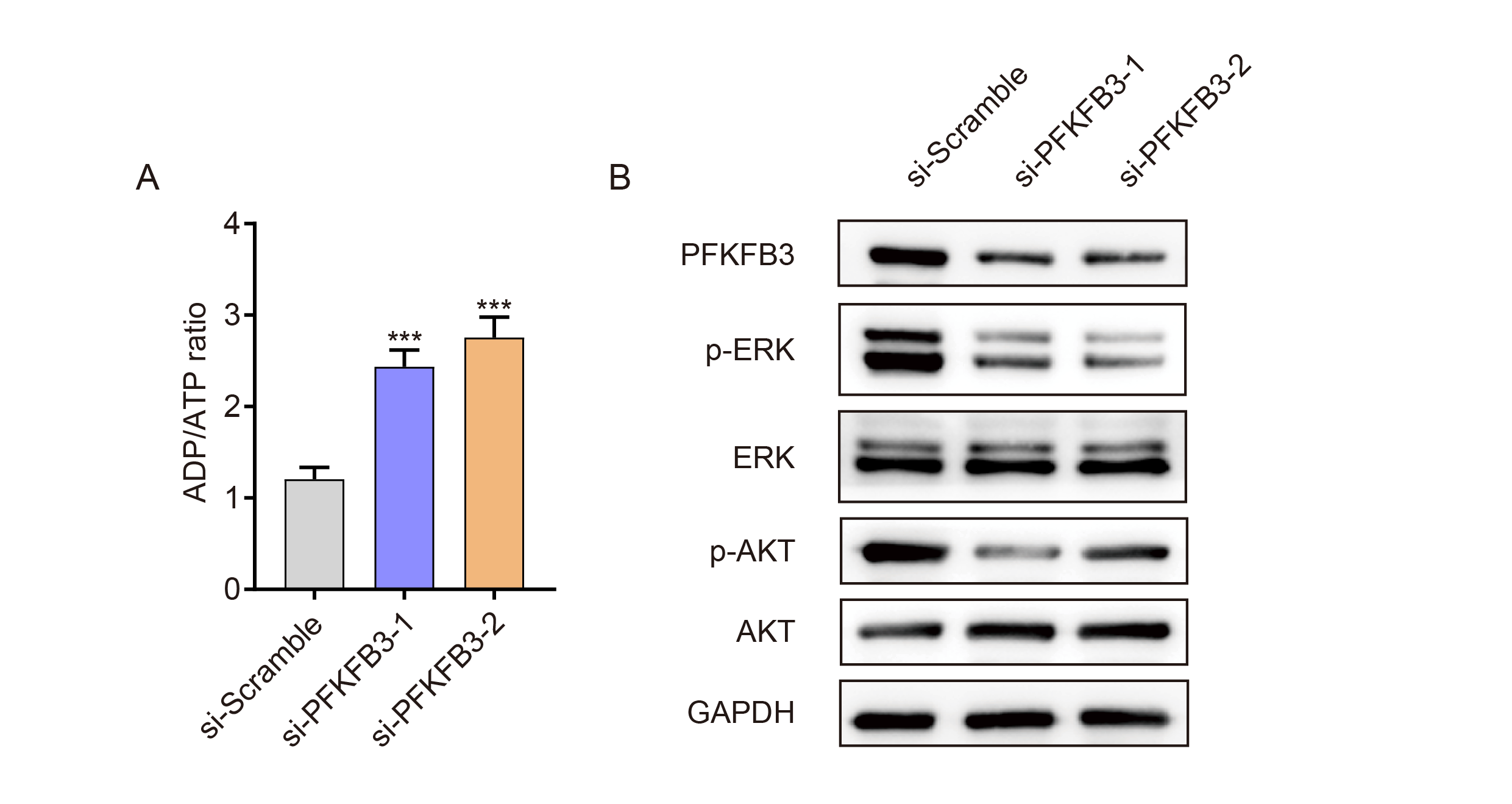

Supplement: Supplementary file 6 — Supplementary Fig.4 [file 41419_2023_5709_MOESM6_ESM.tif]

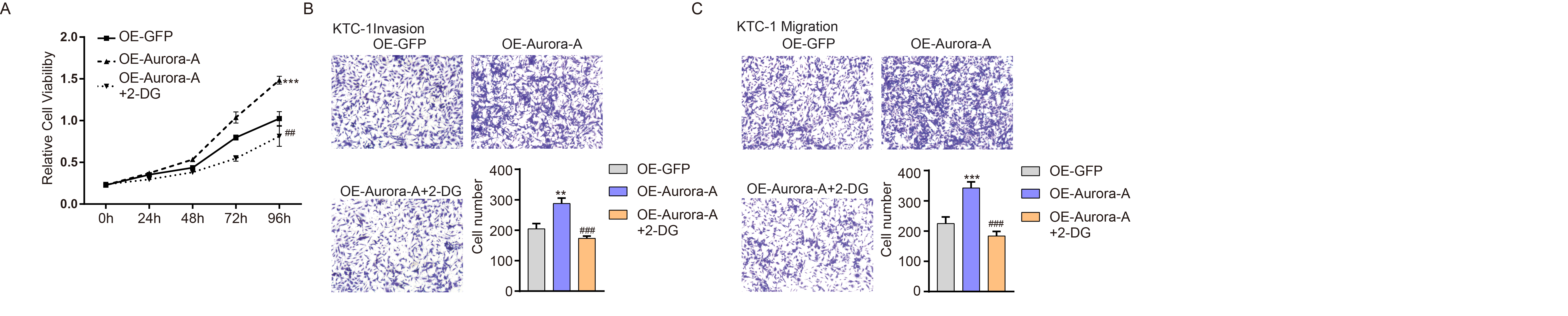

Supplement: Supplementary file 7 — Supplementary Fig.5 [file 41419_2023_5709_MOESM7_ESM.tif]

Fig2

D

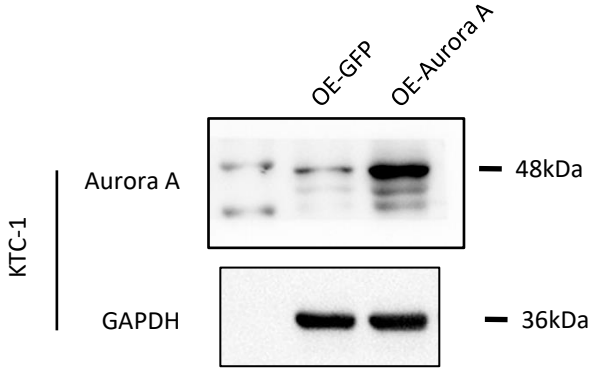

H

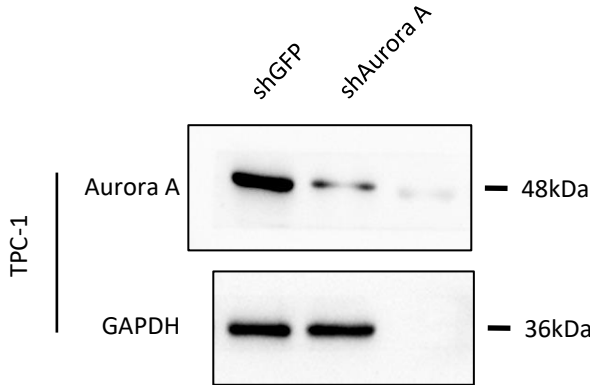

Fig4

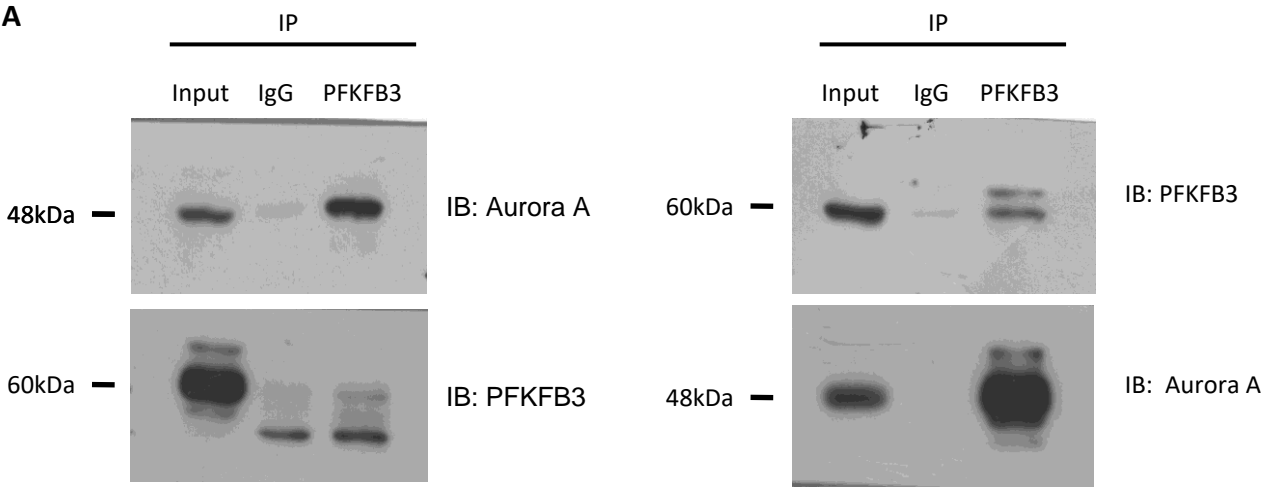

Fig4

B

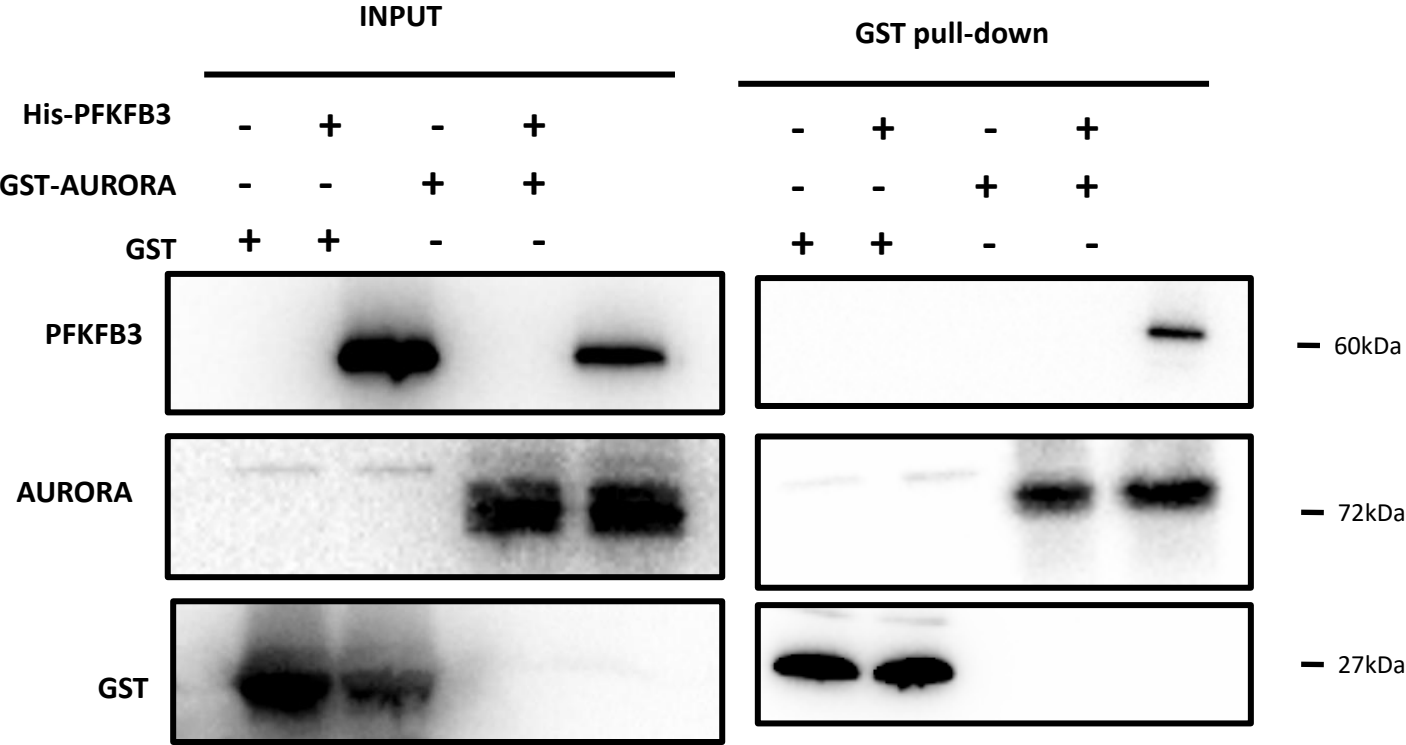

Fig4

c

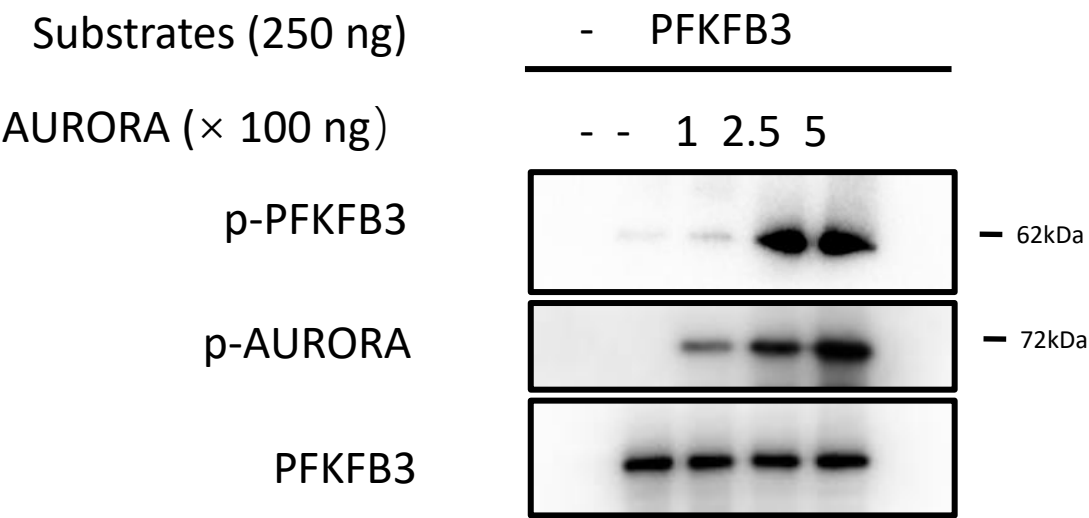

Fig4

D

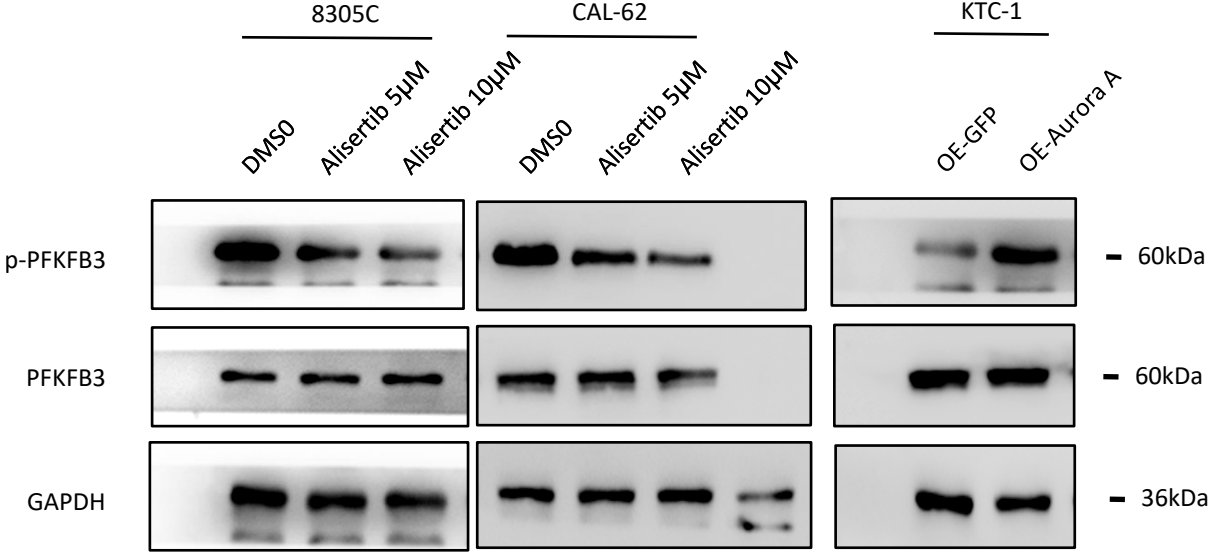

Fig4

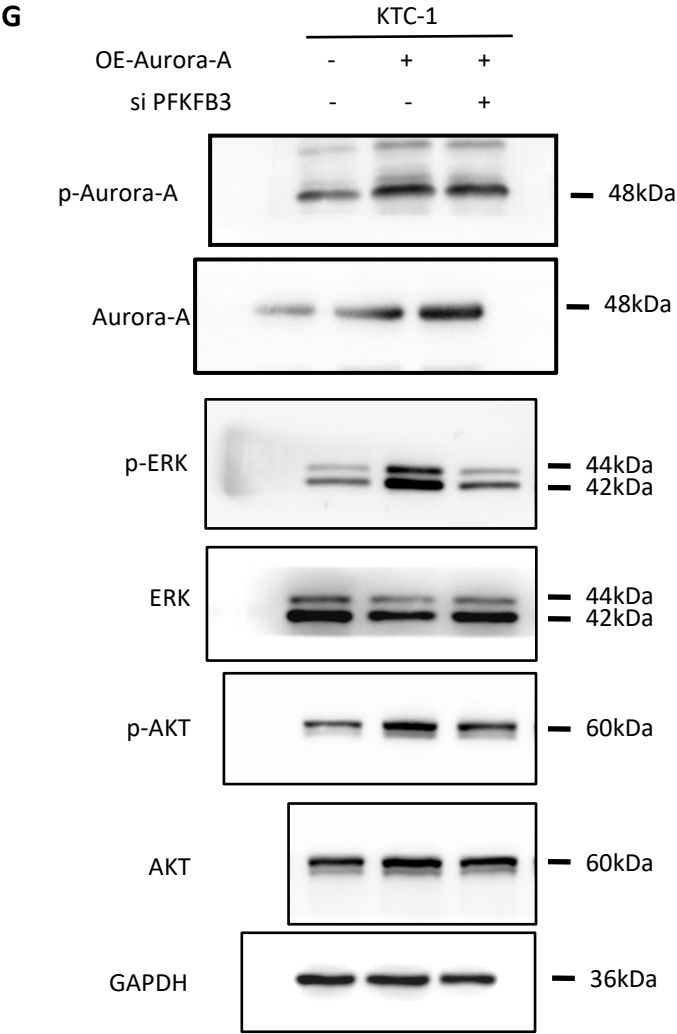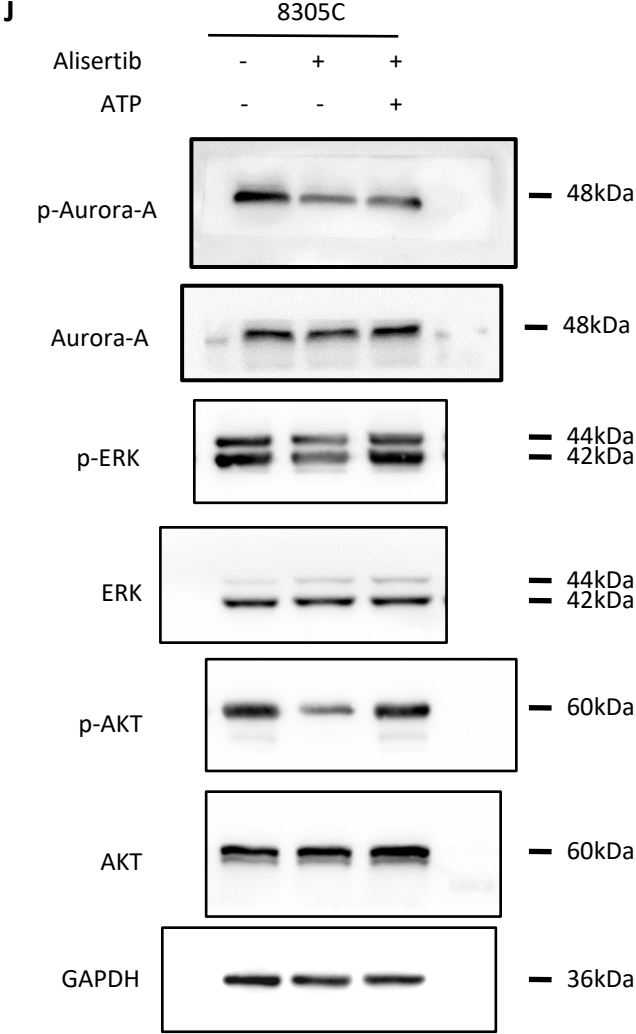

Fig5

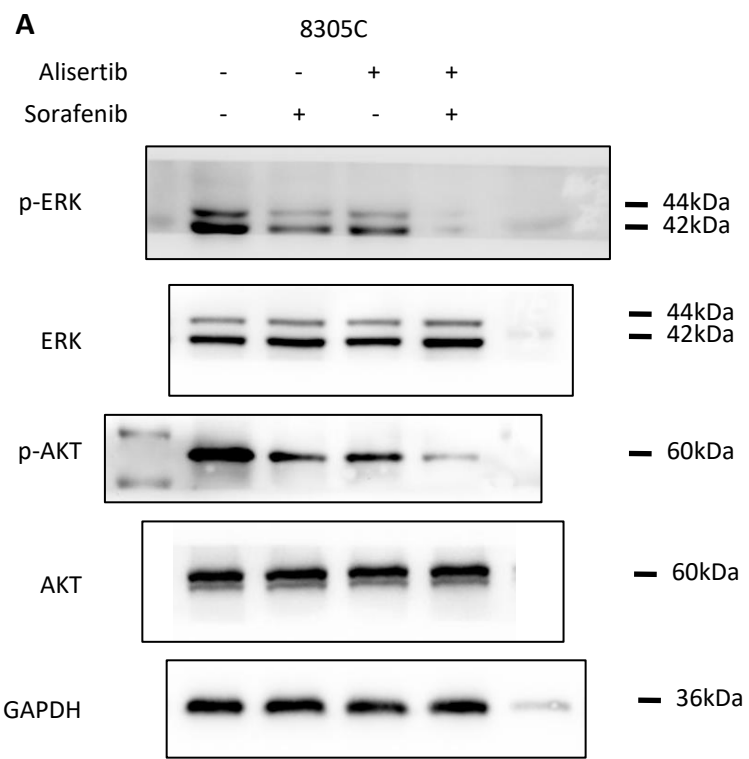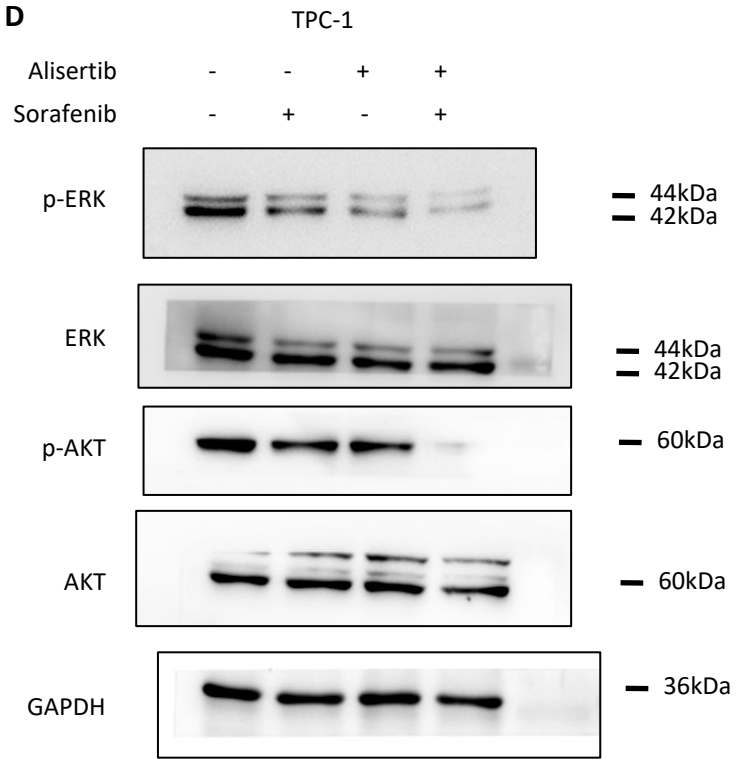

Fig S1

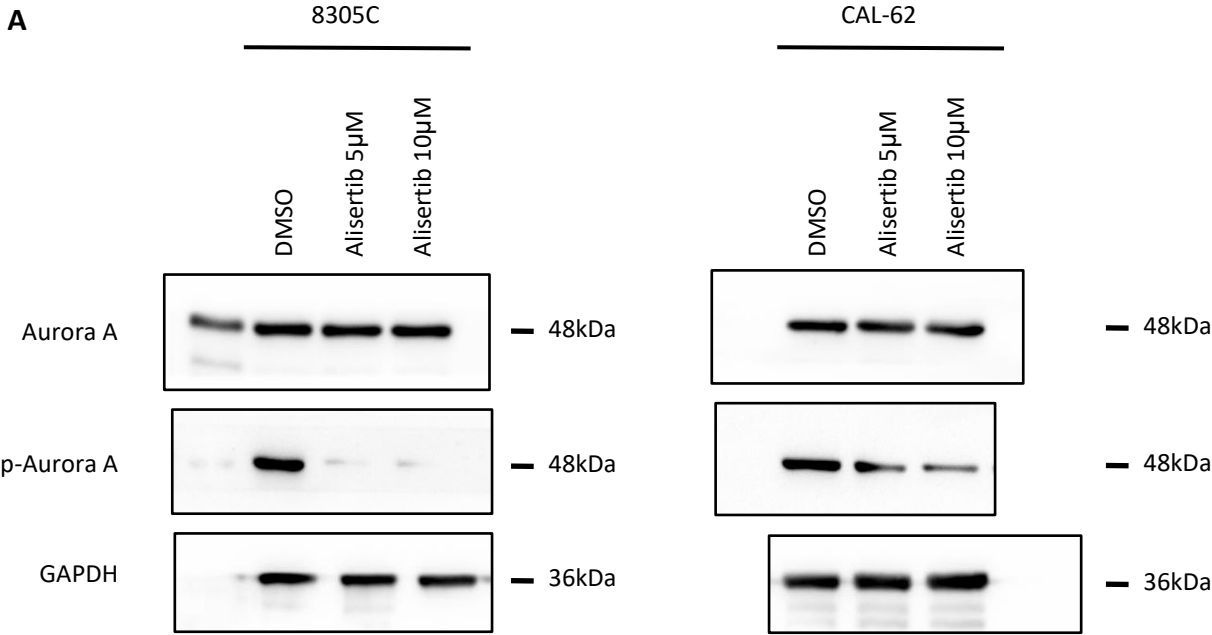

Fig S2

A

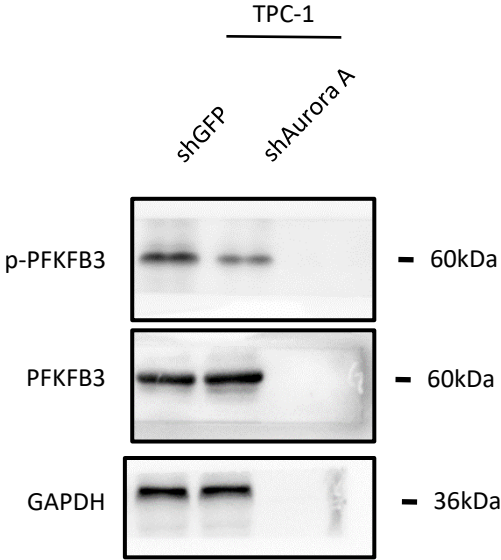

Fig S4

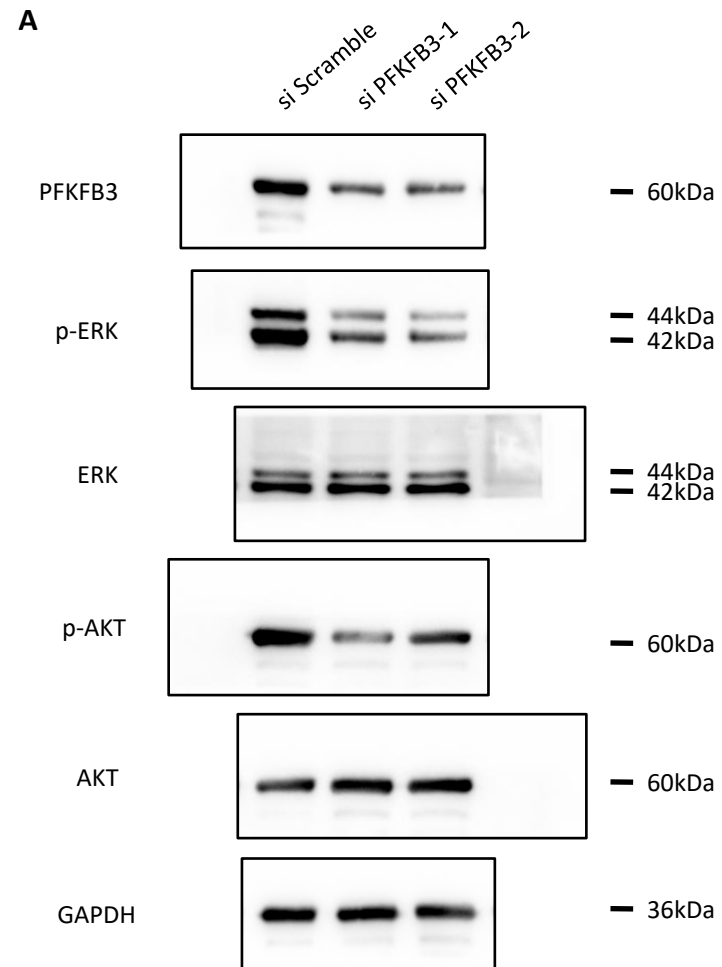

Supplement: Supplementary file 8 — WB original data [file 41419_2023_5709_MOESM8_ESM.pdf]
